# A survey of patient tolerance and satisfaction with capsaicin for neuroproliferative vestibulodynia

**DOI:** 10.1093/sexmed/qfae012

**Published:** 2024-03-27

**Authors:** Isabella Kopits, Jill M Krapf, Chailee Moss, Theodora Mautz, Jess Holloway, Lilliana Starsiak, Sylvia Lorenzini, Andrew T Goldstein

**Affiliations:** The Centers for Vulvovaginal Disorders, Washington, DC 20037, United States; The Centers for Vulvovaginal Disorders, Washington, DC 20037, United States; Department of Obstetrics and Gynecology, The George Washington University, Washington DC, United States; The Centers for Vulvovaginal Disorders, Washington, DC 20037, United States; Department of Obstetrics and Gynecology, The George Washington University, Washington DC, United States; The Centers for Vulvovaginal Disorders, Washington, DC 20037, United States; The Centers for Vulvovaginal Disorders, Washington, DC 20037, United States; The Centers for Vulvovaginal Disorders, Washington, DC 20037, United States; Department of Obstetrics and Gynecology, The George Washington University, Washington DC, United States; The Centers for Vulvovaginal Disorders, Washington, DC 20037, United States; The Centers for Vulvovaginal Disorders, Washington, DC 20037, United States; Department of Obstetrics and Gynecology, The George Washington University, Washington DC, United States

**Keywords:** vestibulodynia, capsaicin, vulvar vestibulitis, dyspareunia, vulvodynia, vulvar pain

## Abstract

**Background:**

Topical capsaicin has been used to treat vulvodynia but has been poorly studied for use in neuroproliferative provoked vestibulodynia (PVD); capsaicin decreases allodynia by blocking vanilloid receptors (TRPV1) on C-afferent nociceptors, but the therapy causes discomfort to the point of intolerance in some patients.

**Aim:**

The present study evaluated tolerability and efficacy of topical capsaicin to treat neuroproliferative PVD.

**Methods:**

Patients with neuroproliferative PVD prescribed 0.025% capsaicin compounded in VersaBase cream were identified through prescription records. Outcome measures included the Female Sexual Function Index (FSFI), the Female Sexual Distress Scale–Revised, and a 22-question questionnaire assessing patient experience and treatment tolerability.

**Outcomes:**

Among tolerant patients, capsaicin significantly decreased vestibular pain, but tolerance was highly variable.

**Results:**

Twenty-five patients responded to the follow-up questionnaire. The average age at presentation was 30 years (range, 18-52 years). Eighty percent of patients tolerated capsaicin application for the full 20 minutes within a median time of 1 to 2 weeks. Of the 16 patients reporting tolerance to 20-minute application, 12 (60%) experienced improvement in vestibular pain. On an 11-point numeric rating scale, the mean pain score was 8.96 and the median score was 10 with first application. Among all participants, 16 (64%) had reduction in pain during treatment. Fifty-six percent of patients would recommend capsaicin as a treatment for vulvar pain. Qualitative content analysis focused on categories of efficacy, value, and feasibility, which indicated that those able to tolerate the treatment experienced improvement while using the medication. The mean Female Sexual Distress Scale–Revised score was 35.96 at baseline compared with 25.09 at follow-up (*P* < .0001). On a numeric rating scale, the mean self-reported vulvar pain score was 8.2 at baseline compared with 5.35 when using capsaicin consistently (*P* < .0001). The mean FSFI pain domain score was 2.45 at baseline compared with 0.98 at follow-up (*P* = .005). While not statistically significant, the mean total FSFI score was 15.44 at baseline compared with 17.84 at follow-up (*P* = .3730).

**Clinical Implications:**

Capsaicin is helpful for some patients with PVD, but thorough counseling is important because of highly variable tolerance.

**Strengths and Limitations:**

Strengths include examination of a poorly studied therapy and inclusion of narrative responses from patients to inform counseling. Limitations include small sample size, retrospective design, and low survey response rate.

**Conclusion:**

Patients should be appropriately selected and thoroughly counseled given high levels of intolerance, but capsaicin therapy may be considered for patients with neuroproliferative PVD who have failed conservative treatments and wish to avoid surgery.

## Introduction

Provoked vestibulodynia (PVD), previously known as vulvar vestibulitis, is the most common form of vulvodynia and comprises pain triggered by vestibular contact^1^^,^^2^. In a population-based study, 64.8% of vulvodynia patients had pain only with provocation at the vestibule.^3^ A subset of PVD is thought to be due to neuroproliferative vestibulodynia,^4^ in which there is an increased density of nociceptive nerve endings in the vestibular mucosa. It has been postulated that this increase in nociceptors occurs either congenitally or secondary to a mast cell–mediated response to an inflammatory process such as a chronic candidiasis or allergic dermatitis.^4^^,^^5^ Currently, the most effective treatment for neuroproliferative PVD is vulvar vestibulectomy.^6^ However, surgical risks, including the loss of vaginal lubrication, scarring, or an increased risk of greater vestibular gland (Bartholin’s) cysts may be unacceptable to some patients. Moreover, vulvar vestibulectomy requires an extensive and potentially costly recovery period, making surgical treatment inaccessible to many. Pharmacotherapy for the disorder has the potential to help patients who prefer alternatives to surgery.

Capsaicin, the chili pepper chemical thought to induce analgesia, is hypothesized to improve vestibular pain for some patients through agonist effects on overexpressed vanilloid receptors (TRPV1) on C-afferent nociceptors.^7^ Studies of topical capsaicin for PVD have had mixed results. Steinberg et al. demonstrated that twelve weeks of daily capsaicin application reduced general vulvar pain and increased frequency of sexual relations among women with PVD.^8^ Murina et al^9^ reported that patients experienced an improvement in vulvar pain while using capsaicin at least twice weekly. Patients showed a mean reduction in pain of 76% after 24 weeks of therapy, although they did not report complete remission, and pain returned with discontinuation of capsaicin therapy.

Because it is associated with significant pain during initial use and has a lack of durable response, capsaicin therapy for PVD has been met with understandable resistance, with even with some clinicians voicing opposition to study of the medication in the literature.^10^ Studies of capsaicin therapy for PVD have also been limited by sample size and inability to blind patients to application.^9^ However, for patients with severe symptoms who cannot or do not want surgery and for whom other therapies have failed, more substantial therapeutic side effects may be acceptable in pursuit of relief. For these patients, guidance on appropriate dosage and duration of treatment are profoundly important, but as yet unavailable in the literature. Qualitative analysis of patient narrative experiences with the medication are a vital part of that guidance, given the subjective and richly varied nature of tolerability. The aim of this study is to further investigate the therapeutic potential of capsaicin cream as treatment for neuroproliferative PVD in terms of tolerability and efficacy. The central question of the project was the following: is capsaicin cream a subjectively tolerable treatment for neuroproliferative vestibulodynia, and if tolerated, is it effective in improving sexual function?

## Methods

Patients who were prescribed 0.025% capsaicin compounded in VersaBase cream for a diagnosis of neuroproliferative PVD between January 2017 and December 2022 were identified through electronic medical records. The diagnosis of neuroproliferative PVD was confirmed through the practice’s electronic medical records by history in conjunction with cotton swab evaluation by trained vulvar pain specialists at a vulvovaginal disorder clinic. Patients were prescribed the medication with instructions to apply 0.5 g of capsaicin cream to the vulvar vestibule, increasing the length of application daily as tolerated with the goal of 20 minutes of application daily for a minimum of 12 weeks. Patients were offered premedication with 2% lidocaine ointment. Following application, patients were counseled to wash the capsaicin cream off with cold water or milk. Patients who were excluded were under 18 years of age, diagnosed with vulvar pruritus but not vestibulodynia, or prescribed capsaicin but never used it.

Eligible participants who used capsaicin for PVD were contacted by email and telephone by a research assistant. All patients had completed a baseline questionnaire that included the Female Sexual Dysfunction Index (FSFI) and the Female Sexual Distress Scale–Revised (FSDS-R) prior to initial consultation. Patients who opted into the study were sent a secure posttreatment questionnaire and gave permission for study staff to include their baseline questionnaire in the research. The posttreatment questionnaire evaluated pain and sexual function before, during, and after capsaicin therapy (Appendix). In addition, the questionnaire included questions about the duration, frequency, and tolerability of participants’ treatment, as well as if they would recommend capsaicin to another patient with similar symptoms. Patients were also asked to describe their experience with capsaicin qualitatively and were given space for narrative responses. This study was approved by the BRANY Institutional Review Board (ID: 21-12-276-865)*.* Electronic or verbal informed consent was obtained from each participant.

### Outcome measures

Female sexual dysfunction (FSD) was measured with the FSFI and FSDS-R. The FSFI is a validated 19-question survey with 6 sections to assess desire, arousal, lubrication, orgasm, satisfaction, and pain. The FSDS-R is a validated 13-question survey that assesses distress associated with inadequate or impaired sexual function.^11^ Pretreatment data were collected via the practice’s baseline electronic survey that includes these instruments. In addition to the validated questionnaires, participants completed a 22-question electronic questionnaire regarding duration, frequency, tolerability, and effectiveness of capsaicin use (Appendix). This questionnaire was developed and reviewed by vulvar specialists familiar with neuroproliferative PVD and treatment with capsaicin.

### Statistical and qualitative analysis

Descriptive statistics were reported as means and percentages. Bivariate analyses with Student *t* tests were conducted to determine the effect of capsaicin therapy. Statistical analysis was conducted in R version 4.2.2 (R Foundation for Statistical Computing) using the {gt_summary} package. Qualitative data on patient experience with capsaicin treatment were analyzed utilizing the clinical-qualitative method for content analysis. The researchers independently and cooperatively completed the following steps: editing material for analysis, floating reading, construction of codes of meaning, general refining of the codes, and the construction of categories, discussion, and validity (Faria-Schutzer et al. 2021)^12^. Full-text responses to the question “What was your experience using capsaicin cream?” were tabulated in a spreadsheet and 3 authors independently reviewed the responses (authors IK, CM, JK). Reports suggesting similar meaning were grouped together and impactful reports were highlighted, focusing on what the patient expressed in each passage. The units of analysis were given codes of meaning and the full text was again reviewed to gather material for analysis under each code. Each participant’s response was reviewed to refine and expand the codes. The collated data were then categorized in relation to the research question, ensuring that categories were exhaustive and mutually exclusive. Representative and rich quotes were extracted for each category and then reviewed by authors and a patient who experienced the therapy to ensure validity.

## Results

**Figure 1:**
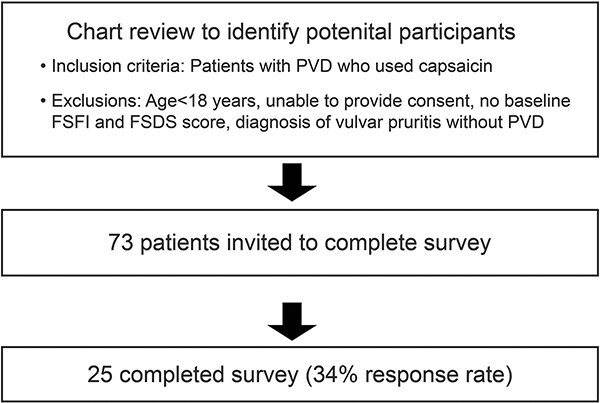
Flow chart of exclusion criteria showing 73 initial potential participants with 25 subsequent completed surveys.

Seventy-three patients were identified as eligible for participation based on filling a capsaicin prescription in the study period. Of those, 25 patients responded to the follow-up questionnaire with a response rate of 34% (Figure 1). The average age at presentation was 30 years (range, 18-52 years). Self-reported ethnicity of the study population was 76% White, 4% Latina, 4% Asian, 4% Mixed, and 4% Middle Eastern, with no response from 8% of participants (Table 1). Eighty-eight percent of patients presented with at least 1 other diagnosis associated with genitopelvic pain at the time of initiating capsaicin. Despite capsaicin therapy, 8 (32%) patients went on to have a vulvar vestibulectomy; their self-reported pain scores before surgery were included, but postsurgical responses were not reported. At the time of the survey, 3 patients reported current use of the medication, while 4 patients had discontinued use in the last year. The remaining 18 patients had not used the medication for over a year at the time that they completed the survey.

**Table 1:** Demographics.

Age, y	30.7 ± 7.91
**Race/ethnicity**	
White	19 (76)
Latina	1 (4)
Asian	1(4)
Middle Eastern	1(4)
Unknown	2(8)
**Additional diagnoses**	
Hypertonic pelvic floor muscle dysfunction	18(72)
Pudendal neuralgia	3(12)
Clitorodynia	1(4)
Other diagnoses^a^	5 (20)

Values are mean ± SD or n (%).

a Recurrent *Candida*, vaginismus, desquamative inflammatory vaginitis, lichen simplex chronicus.

**Figure 2:**
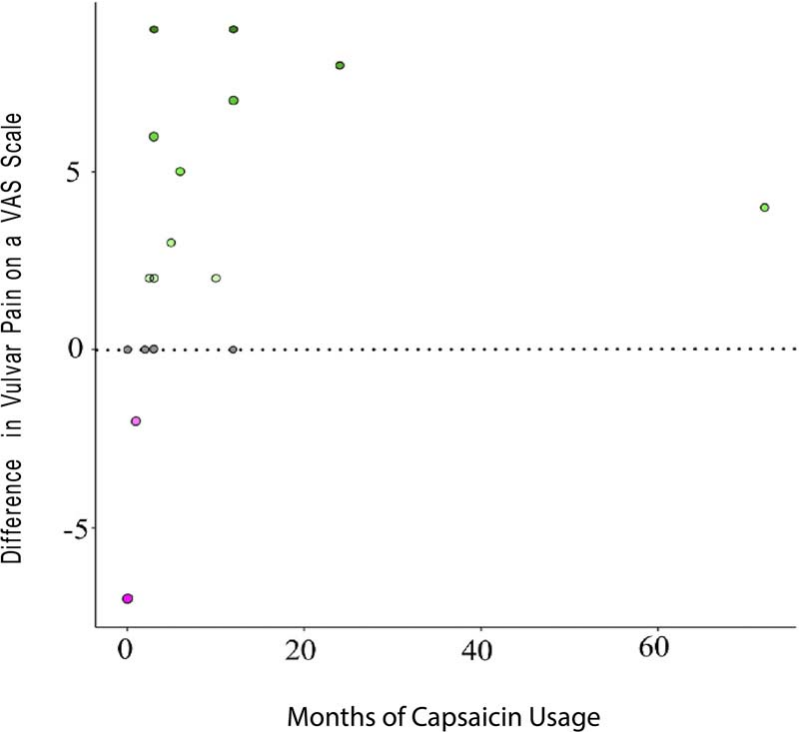
Change in vulvar pain is depicted on a numeric rating scale (NRS). The dotted line represents no change. Below the zero line, there is a gradient that shows worsening pain; above the zero line shows a gradient representing an improvement in vulvar pain on an NRS. Those who experienced an improvement in pain used capsaicin for a longer duration.

Patients reported significantly less vulvar pain during capsaicin therapy (Table 2). On an 11-point NRS, the mean self-reported vulvar pain score was 8.2 at baseline compared with 5.35 when using capsaicin consistently (*P* < .0001). Sixteen (64%) patients reported a decrease in vulvar pain while using capsaicin consistently. Among those who could tolerate capsaicin application for the full 20 minutes, patients reported a mean 44% decrease in vulvar pain while using capsaicin consistently (*P* < .0001). Among those who did not undergo a vestibulectomy, mean vulvar pain after finishing capsaicin therapy or decreasing to a maintenance dose was 2.94 on an NRS (*P* < .0001). Patients who had a good response to capsaicin tended to use the therapy for a longer duration (Figure 2).

**Table 2:** Main outcome measures.

Main outcome measure	Baseline	After capsaicin	*P* value^a^
Self-reported vulvar pain	8.20	5.35	<.001
FSDS-R	35.96	25.09	<.001
FSFI total	15.44	17.84	.373
Desire	2.95	2.69	.407
Arousal	2.96	2.88	.881
Orgasm	2.72	2.66	.919
Satisfaction	2.63	3.71	.020
Pain	0.98	2.46	.005

a Welch 2-sample *t* test.

**Figure 3:**
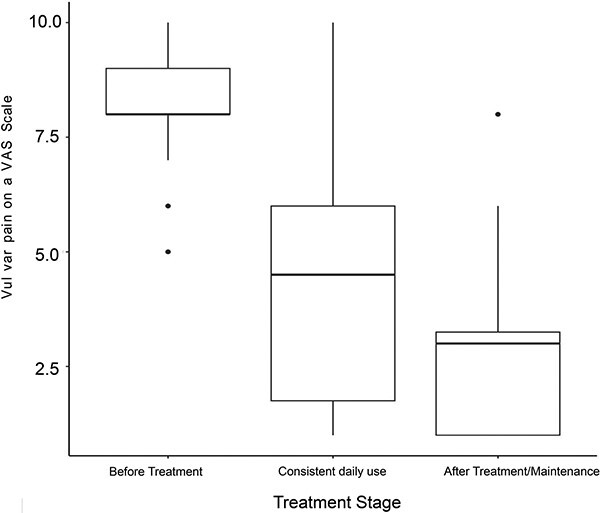
Vulvar pain scores for patients who did not subsequently undergo vestibulectomy. The figure shows a box plot with decreasing scores over time.

In addition to decreasing self-reported vulvar pain, capsaicin therapy also decreased sexual dysfunction. The mean FSDS-R score was 35.96 at baseline compared with 25.09 at follow-up (*P* < .0001). While not statistically significant, the mean total FSFI score was 15.44 at baseline compared with 17.84 at follow-up (*P* = .3730). However, mean FSFI pain domain score was 0.98 at follow-up compared with 2.45 at baseline (*P* = .005). For patients who did not subsequently undergo vestibulectomy, NRS scores decreased during capsaicin therapy as well as after capsaicin therapy cessation or maintenance (Figure 3).

On an 11-point NRS, the mean pain score while initially applying capsaicin was 8.96, and the median was 10 with first use. While 64% of patients found that capsaicin improved their vulvar pain, 56% of patients would recommend capsaicin as a treatment for vulvar pain. No patients reported complete resolution of their vulvar pain after topical capsaicin therapy using self-reported pain, FSDS-R, or FSFI scores, but 2 patients reported complete remission subjectively.

### Treatment regimen

After consistent daily usage, 80% of patients were able to tolerate 0.5 g capsaicin application for the full 20 minutes. The median amount of time required to increase tolerance to the full 20 minutes was 1 to 2 weeks of consistent daily usage. Washing the cream off with cold water was helpful for tolerating capsaicin application for 72% of patients, and 8% of patients found applying a topical anesthetic such as lidocaine to be helpful. Five patients reported that meditation or distraction helped them tolerate capsaicin. Additionally, 24% of patients reported that applying an ice pack to the vulva helped relieve the pain of capsaicin application.

### Qualitative content analysis

Three categories were developed from qualitative content analysis: efficacy, value, and feasibility. Experience of efficacy with capsaicin was varied (Table 3). Patients who were able to tolerate capsaicin reported feeling “grateful” and “satisfied” with the treatment. Two patients described the treatment as “life-changing,” and 3 reported that capsaicin was “the only thing that worked” for their pain. Two patients reported that capsaicin exacerbated their vulvar pain; both discontinued use within 2 weeks of initiation. Emotional distress was reported among patients who were not able to tolerate initial capsaicin application. Patients able to tolerate the first application and continue treatment expressed a theme of value: improved pain and function despite the negative aspects of treatment (Table 3). In regard to feasibility, patients expressed annoyance and discomfort with repeated application and need for continued application to prevent pain from recurring (Table 3). Three patients were still using a maintenance dose of capsaicin for continued pain management a few times per month at the conclusion of the study. Four patients reported that their pain returned when they stopped using capsaicin, but 4 reported that their pain never returned after capsaicin therapy. Two patients who went on to have a vulvar vestibulectomy endorsed capsaicin therapy as a good therapeutic option for short-term pain management prior to definitive surgical management.

**Table 3:** Patient comments on capsaicin experience.

Efficacy	“It was life-changing—the only thing that worked after two years of pain, during which I tried many other treatments. I have been completely pain-free for nearly 10 years.” “I have been using it for several years and have felt somewhat successful in addressing my pain. I can now have intercourse with minimal pain.” “The cream I think was—when nothing else worked (multiple botox injections, topical lidocaine, valium suppositories, pelvic floor PT [physical therapy] for years)—the only thing that I believe helped move the needle towards my being mostly pain-free…I never thought I’d be pain-free again, so I’m incredibly thankful! “I wish it had worked for me—I clearly became tolerant but it just didn’t change the pain of intercourse.” “It didn’t help me at all. It caused more discomfort once I was applying it on a daily basis than it did any good.”
Value	“I was very thankful for capsaicin. Although it was uncomfortable to use. I would rather have my vagina feel like it is burning during the 20 minutes a day rather than not use it and feel my vagina burning in pain all day long.” “It was extremely painful but very worthwhile. I’m glad I did it.” “It hurts as hell but it changed my life to the better.” “It was a serious discomfort when first using it. I cried the first time not just because of the pain but I hated that I had to go these lengths to do this to find relief. But it was incredible when I finally [was] able to have sex with a partner I had for a while.” “I pushed through the pain but the result was my that happy, relaxed pelvic floor turned hard as a rock again…My body that I worked so hard to relax would respond to touch down there by recoiling out of fear from that horrible pain induced by the capsaicin.” “It was awful. Painful, traumatizing, and not helpful…Of course it must for some people—but for the amount of pain it causes, is it even worth it? When you are as desperate as I was, women will try anything.”
Long-term feasibility	“The cream works, but it’s too hard to keep doing it every night, so I spaced it out to a couple times a week. Many times that works for several weeks, but then I have episodes of pain again…and I then have to go back to applying the cream several nights in a row…it’s annoying to keep applying it over time, and it’s hard to find a balance with spacing the applications out.” “It hurt way too much to keep going. It made me feel weak, like I couldn’t handle it. I wonder if I should try it again with more determination this time.” “For me it was a temporary solution. It helped me have intercourse at the time but once I stopped using it all the pain came back with no improvement.” “Overall, it didn’t cure my pain, it only helped it a very small amount. The vestibulectomy surgery was what actually cured it.”

## Discussion

Patients had a markedly broad range of responses to capsaicin therapy. Capsaicin is one of only a few nonsurgical treatments available for neuroproliferative PVD. This survey assessment of patient experiences revealed that consistent capsaicin application significantly decreased vulvar pain and sexual distress in those patients who were able to tolerate the therapy. This is in keeping with prior research that habitual capsaicin exposure decreases the release of substance P from nociceptors.^7^ It is also consistent with studies of the medication that show it can be effective for vulvar vestibular pain.^8^^,^^9^^,^^13^ Other therapies, including gabapentinoids, tricyclic antidepressants, selective serotonin reuptake inhibitors, and serotonin and norepinephrine reuptake inhibitors remain first-line options with lower potential for detrimental pain side effects.^6^^,^^14^ There were no statistically significant improvements in overall sexual function with capsaicin therapy based on the FSFI; however, there was a significant decrease in sexual distress measured by the FSDS-R.

Prior studies have not included narrative responses of patient experience, and we feel that this inclusion greatly adds to the ability for clinicians and patients to appropriately consider the range of tolerability and efficacy prior to initiation of the medication. In this study, several patients subjectively reported that the medication markedly improved their lives and sexual function. However, there were patients who reported severe pain and even significant harm from the therapy. Narrative responses from patients who used capsaicin included themes related to the efficacy, value, and long-term feasibility of the therapy and reflected variability in experience in the quantitative data.

Daily application of capsaicin 0.025% cream for 20 minutes for a minimum of 12 weeks followed by a maintenance regimen was effective for improving PVD in tolerant patients, and some patients reported that this was useful in improving tolerability of their condition as a bridge to future surgery. Based on the findings of the current and previous studies, we recommend thorough counseling prior to prescribing capsaicin as a therapy for neuroproliferative PVD. Capsaicin therapy should be presented as an option with risks, benefits, and alternatives so that patients may make an informed decision in care. Capsaicin appears to be effective in patients that are able to tolerate the initial application of the medication and are able to increase application as prescribed. Techniques to improve tolerability include application of the cream for a short time period initially and increasing length of application nightly, washing the cream away immediately with a cold liquid, and having adequate expectations on the experience of application. Capsaicin therapy will likely need to be continued to sustain pain relief and function. Capsaicin may be utilized as an alternative to surgery or a temporary measure while preparing for surgery.

The limitations of this study include a relatively small sample size and low response rate. It is difficult to know if the patients who did not respond would have better or worse responses than the respondents, but given the significant dearth of data regarding capsaicin therapy and nonsurgical management of primary PVD in general, we feel that the data presented here are still quite valuable. Moreover, this study relies on the FSFI to assess sexual function. The FSFI score depends on the respondents having had sex within the past 4 weeks. Three patients were not sexually active but reported decreased vulvar pain. In a 2005 validity study of the FSFI, the cutoff for sexual dysfunction was determined to be a score of 26.55 out of 36. While total FSFI scores were not significantly lower after capsaicin therapy, only 5 patients were above the threshold for sexual dysfunction. Given that 88% had another diagnosis associated with dyspareunia, the FSFI and FSDS-R scores may not show the true effect of capsaicin therapy on female sexual function. This study also uses subjective ratings of vulvar pain before and after treatment. While we believe that subjective assessment of pain is useful, several patients filled out the questionnaire months or years after trying capsaicin; this time lag may introduce recall bias.

The primary strength of this study is including only patients with presumptive neuroproliferative vestibulodynia as the primary vulvar pain diagnosis (the diagnosis of neuroproliferative PVD was confirmed by histopathology in 100% of women who eventually discontinued capsaicin therapy and had vulvar vestibulectomy). This allows evaluation of a treatment targeted to decrease nociceptive pain in patients with nociceptive pain conditions, providing a more accurate evaluation of efficacy of this specific medication in patients with dyspareunia. Additional strengths of the study include examination of a therapeutic modality that has been poorly characterized and the large amount of data gathered about individual patient experiences. The narrative responses patients provided are particularly informative for clinicians and patients; the range of their responses provides a richer understanding of the breadth of the patient experience with this medication and can inform best practices with choosing the most appropriate patients for this treatment, providing specific application instructions and setting expectations to increase tolerability and adherence.

Future work could be directed toward elucidating the genetic and neurologic underpinnings of the heterogeneity of patient response to this therapy. It is unknown why 3 patients had complete pain resolution after stopping the medication. There is also no clear practice regarding maintenance dosing vs a single course of therapy, and this warrants further investigation. Efforts should also focus on alternative nonsurgical treatments for presumed neuroproliferative PVD that are more tolerable and provide definitive treatment.

## Conclusion

Capsaicin cream is a promising treatment for neuroproliferative PVD. When patients have failed more conservative treatments and do not wish to pursue surgery, capsaicin can provide pain relief, temporarily or in the long term. Proper counseling regarding application and measures to mitigate discomfort is essential.

## Supplementary Material

Appendix1-studyquestionairre_qfae012

## References

[1] Bornstein J, Goldstein AT, Stockdale CK, et al. 2015 ISSVD, ISSWSH, and IPPS consensus terminology and classification of persistent vulvar pain and vulvodynia. J Low Genit Tract Dis. 2016;20(2):126–130. 10.1097/LGT.0000000000000190 27002677

[2] Nagandla K, Sivalingam N. Vulvodynia: integrating current knowledge into clinical practice. Obstet Gynaecol. 2014;16(4):259–267. 10.1111/tog.12130

[3] Reed BD, Harlow SD, Sen A, et al. Prevalence and demographic characteristics of vulvodynia in a population-based sample. Am J Obstet Gynecol. 2012;206(2):170.e1–170.e9. 10.1016/j.ajog.2011.08.012 PMC3779055 21963307

[4] Lev-Sagie A, Witkin SS. Recent advances in understanding provoked vestibulodynia. F1000Res. 2016;5:2581. 10.12688/f1000research.9603.1 27853523 PMC5089138

[5] Pukall CF, Goldstein AT, Bergeron S, et al. Vulvodynia: definition, prevalence, impact, and pathophysiological factors. J Sex Med. 2016;13(3):291–304. 10.1016/j.jsxm.2015.12.021 26944461

[6] Goldstein AT, Pukall CF, Brown C, Bergeron S, Stein A, Kellogg-Spadt S. Vulvodynia: assessment and treatment. J Sex Med. 2016;13(4):572–590. 10.1016/j.jsxm.2016.01.020 27045258

[7] Frias B, Merighi A. Capsaicin, nociception and pain. Molecules. 2016;21(6):797. 10.3390/molecules21060797 27322240 PMC6273518

[8] Steinberg AC, Oyama IA, Rejba AE, Kellogg-Spadt S, Whitmore KE. Capsaicin for the treatment of vulvar vestibulitis. Am J Obstet Gynecol. 2005;192(5):1549–1553. 10.1016/j.ajog.2004.10.626 15902156

[9] Murina F, Radici G, Bianco V. Capsaicin and the treatment of vulvar vestibulitis syndrome: a valuable alternative? MedGenMed. 2004;6(4):48 PMC1480562 15775875

[10] Akel R, Cohen CE, Fuller C. Caution with topical capsaicin. Clin Exp Dermatol. 2020;45(6):739. 10.1111/ced.14226 32215935

[11] Carpenter JS, Reed SD, Guthrie KA, et al. Using an FSDS-R item to screen for sexually related distress: a MsFLASH analysis. Sex Med. 2015;3(1):7–13. 10.1002/sm2.53 25844170 PMC4380908

[12] Faria-Schützer, et al. Seven steps for qualitative treatment in health research: the Clinical-Qualitative Content Analysis. Cien Saude Colet. 2021;26(1):265-274. 10.1590/1413-81232020261.07622019 33533847

[13] Sadownik LA . Etiology, diagnosis, and clinical management of vulvodynia. Int J Womens Health. 2014;6:437–449. 10.2147/IJWH.S37660 24833921 PMC4014358

[14] Bachman GA, Brown CS, Phillips NA, Rawlinson LA, You X, et al. Effect of gabapentin on sexual function in vulvodynia: a randomized, placebo-controlled trial. Am J Obstet Gynecol. 2019;220(1):89.e1–89.e8. 10.1016/j.ajog.2018.10.021 PMC6310649 30365922

