# Supplementary material for: A survey of patient tolerance and satisfaction with capsaicin for neuroproliferative vestibulodynia
Source: Sex Med. 2024 Mar 27;12(1):qfae012. doi: 10.1093/sexmed/qfae012 (PMC10971571; doi:10.1093/sexmed/qfae012)
Supplement: Appendix1-studyquestionairre_qfae012 [file appendix1-studyquestionairre_qfae012.docx]

Appendix 1 - Capsaicin Study Questionnaire

1. Prior to using capsaicin cream, how would you rate your vulvar pain (on a scale from 0 - 10, where 0 is no discomfort and 10 is extreme discomfort)?
2. How long did you use capsaicin cream? Please respond in days, weeks, months, or years. [free text response]
3. How many times did you use capsaicin cream?
4. Once
5. A few times (2 - 5 times)
6. Several times (5 - 10 times)
7. Many times (>10 times)
8. How frequently did you use capsaicin cream?
   1. Daily
   2. More than once per day
   3. Every other day
   4. A few times per week
   5. Weekly
   6. Other [free text]

1. How long did you typically leave capsaicin cream on your vulva before washing it off?
   1. <1 minute
   2. 1-5 minutes
   3. 6-10 minutes
   4. 11-20 minutes
   5. 21-30 minutes
   6. >30 minutes
2. Were you able to increase capsaicin cream application to the full 20 minutes?
   1. Yes
   2. No
3. If yes, how long did it take for you to increase tolerance to the 20 minute application of capsaicin cream (e.g. 4 weeks of daily use)? [free text]
4. If no, how many minutes were you able to tolerate capsaicin cream at most (e.g. 7 minutes)? [free text]
5. When you first tried capsaicin cream, how uncomfortable was applying it (on a scale from 0-10, where 0 is no discomfort and 10 is extreme discomfort)?
   1. 1
   2. 2
   3. 3
   4. 4
   5. 5
   6. 6
   7. 7
   8. 8
   9. 9
   10. 10
6. Did the discomfort when applying capsaicin cream to your vulva improve over time?
   1. Not at all – I always felt the same discomfort while applying capsaicin
   2. A little improvement over time
   3. A lot of improvement over time
   4. Complete improvement over time – I no longer felt any discomfort applying capsaicin
   5. I discontinued use of capsaicin after only a few applications because it was so uncomfortable to apply
   6. I never felt discomfort when applying capsaicin
7. What did you find helpful in making application of capsaicin cream more tolerable?
   1. Washing the cream off with cold water
   2. Washing the cream off with cold milk
   3. Applying ice to your vulva
   4. Meditation or relaxation techniques
   5. Using a timer
   6. Applying a vulvar moisturizer
   7. Applying topical numbing medication (such as lidocaine cream) first
   8. Other [free text]
8. When you were using capsaicin cream consistently, how did you rate your vulvar pain (on a scale from 0-10, where 0 is no discomfort and 10 is extreme discomfort)?
   1. 1
   2. 2
   3. 3
   4. 4
   5. 5
   6. 6
   7. 7
   8. 8
   9. 9
   10. 10
9. Did you have any side effects from using capsaicin cream? [free text]
10. How long has it been since you used capsaicin cream?
    1. I am currently using capsaicin cream
    2. Less than 1 month
    3. 2-5 months
    4. 3-6 months
    5. 6-12 months
    6. Over 1 year
11. How would you rate your vulvar pain now (on a scale from 0-10, where 0 is no discomfort and 10 is extreme discomfort)?
    1. 1
    2. 2
    3. 3
    4. 4
    5. 5
    6. 6
    7. 7
    8. 8
    9. 9
    10. 10
12. If you used capsaicin cream in the past but have discontinued it, how would you describe its effects on your overall vulvar discomfort during use and after stopping, including a time course? [free text]
13. Would you recommend capsaicin cream as a treatment for others suffering from vulvar pain similar to yours?
    1. Yes
    2. No
    3. Other [Free text]
